# Novel Efficient Reduction Route for Magnesium Production Using Silicothermic Process

**DOI:** 10.3390/ma15176009

**Published:** 2022-08-31

**Authors:** Yongqiang Chen, Gengpeng Mai, Yusi Che, Jilin He

**Affiliations:** 1 School of Material Science and Engineering, Zhengzhou University, Zhengzhou 450001, China; 2 Henan Province Industrial Technology Research Institute of Resources and Materials, Zhengzhou University, Zhengzhou 450001, China

**Keywords:** magnesium production, silicothermic method, mass transfer enhancement, porous pellet

## Abstract

A novel efficient reduction route was developed for preparing porous pellets to enhance mass transfer during magnesium production, which can improve the reactivity of pellet reaction to improve the reduction efficiency. A porous pellet precursor was prepared at 150 MPa using NH_4_HCO_3_ as a pore-forming agent, and the reaction characteristics of the pellets with 0, 5%, 10%, 20%, and 30% pore-forming agents were measured under a high vacuum of approximately 10 Pa heat-treated from 100 °C to 1400 °C. The results showed that the instantaneous maximum reduction rate first increased and then decreased with the increase in pore-forming agents. When the reduction conversion was 80%, the reduction efficiency of pellets with 5% pore-forming agent was 36% greater than that without pore-forming agent pellets. When the reduction conversion was 90%, the reduction efficiency of pellets with 5% pore-forming agent was 29% greater than that without pore-forming agent pellets. The results indicate that the diffusion rate of magnesium vapor in pellets is significantly increased; the time of chemical reaction reaching equilibrium is shortened; the chemical reaction rate and the magnesium production efficiency are increased by adding a proper ratio of NH_4_HCO_3_ compared to that obtained without NH_4_HCO_3_ at the identical reduction temperature.

## 1. Introduction

Magnesium is the lightest of all metals used as the basis for constructional alloys. Magnesium also has good electromagnetic interference shielding. It is these properties that give magnesium its broad development prospects [1,2,3]. Magnesium can be used in automotive [4], aerospace [5], military equipment [2], medical [6], 3D products [7], electronics, mobile devices, computer components, and other fields [8,9,10,11,12,13], and is considered to be among the “21st Century Green Engineering Materials” [12,14]. At present, the production methods of magnesium are mainly thermal reduction and electrolysis. Because the Pidgeon process has the advantages of short process flow, low investment, and low cost, since 2007, 80% of the world’s primary magnesium has been produced in China by the Pidgeon process [15,16].

In the last decade, due to high energy consumption, pollution, high production costs, and the lack of solutions to these problems, the development of the magnesium industry has been stagnant. However, developing a low-cost new green magnesium production process is the key to opening the downstream application market for the magnesium industry [17]. Consequently, magnesium production using carbon instead of silicon has become an important research direction due to its environmental protection and low consumption advantages. Professor Dai’s team at Kunming University of Science and Technology has studied magnesium production by carbothermal process in vacuum for a long time [18,19]. They investigated the behavior of CaF_2_ and magnesium and carbon monoxide vapor in reduction process in vacuum [20,21]. Nevertheless, the reversible reaction seriously affected the yield of magnesium, which made it difficult to realize industrial production by the carbothermal method [22]. The Commonwealth Scientific and Industrial Research Organization has succeeded in using a Laval nozzle to coagulate supersonic magnesium vapor in the lab, which could inhibit the reaction between magnesium vapor and carbon monoxide [23,24,25]. Fu et al. [26,27] proposed a novel short process for magnesium production by silicothermic reduction of prepared dolomite pellets, in which calcination and reduction were performed in one retort. Zhang et al. [17] developed a new efficient one-step technical method for magnesium production, which directly uses dolomite and ferrosilicon to make pellets. However, the pellet preparation is a difficult problem to solve. Che et al. [28,29] investigated magnesium production using the silicothermic process under argon flow, which could be produced continuously. At present, there is no report on the successful industrial application of these methods, but they provide a good idea and direction for the magnesium metallurgy research field.

In the Pidgeon process, magnesium vapor is extracted from pellets by a thermal reductive reaction at 1200 °C under a high vacuum of 10 Pa [30,31,32], and the reduction energy consumption is approximately 3.0 tce/tMg [33,34]. In this paper, the factors affecting mass transfer on magnesium production using the Pidgeon process, the current critical method, were evaluated. We found that the operating distance between the raw material and reducing agent decreased after the powder was pressed into pellets, which was conducive to the mutual diffusion of solid substances, but it also increased the resistance of magnesium vapor escaping from the pellets. To solve this problem, a novel method to enhance mass transfer during magnesium production by preparing porous pellets was proposed. Porous pellet precursors with different ratios of the pore-forming agent were prepared by using ammonia bicarbonate as a pore-forming agent, and the effect of porosity on the mass transfer of porous pellets was evaluated by measuring the reaction characteristics of porous pellets under high temperature and vacuum conditions.

## 2. Materials and Experimental Procedures

### 2.1. Materials

In this study, the raw materials are the same as those in the traditional Pidgeon process, and the dolomite is from Wutai Mountain in Shanxi Province. Calcined dolomite (CaO·MgO) is used as the starting material; 75% ferrosilicon (Fe·Si) alloy is used as the reducing agent; fluorite (CaF_2_) is used as the mineralizing agent; ammonium bicarbonate (NH_4_HCO_3_) is used as the pore-forming agent. The purity of NH_4_HCO_3_ is 99.9%, and other material compositions are shown in Table 1.

### 2.2. Preparation of Porous Pellet Precursors

The following points should be noted when adding the pore-forming agent to the pellets to increase the porosity. First, the pore-forming agent itself does not react with all the materials in the magnesium production process, and the gas released during the pore-forming process does not react with the raw materials. Second, the decomposition or phase transformation temperature of the pore-forming agent should be lower than 1000 °C, which is obviously staged with the main reduction reaction, and the pore-forming process is completed before the production of magnesium vapor. Third, the gas produced during the pore-forming process can escape from the pellet without destroying the overall structure of the pellet or even breaking it.

**Figure 1:**
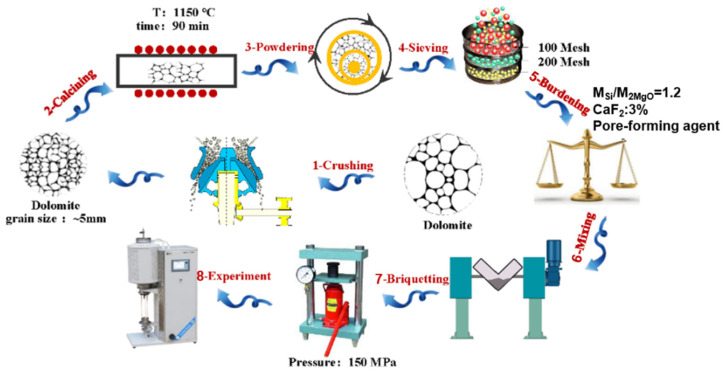
The craft of pellet preparation.

The pellet preparation process is the same as traditional magnesium production using the silicothermic method, as shown in Figure 1.

**Figure 2:**
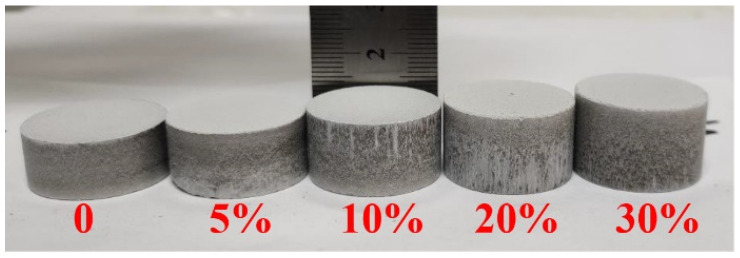
The pellet was formed by pressing after adding the pore-forming agent.

(1) A certain amount of dolomite with a uniform particle size was calcined in a muffle furnace at 1150 °C for 90 min; then, calcined dolomite with 30% activity and a 47.4% burning loss rate was obtained.
(2) Ground calcined dolomite and 75% ferrosilicon were ground through a 100-mesh screen, and the ingredients were prepared according to the molar ratio of M_Si_:M_2MgO_ = 1.2. Fluorite addition was carried out according to a mass ratio of 3%, then different ratios of ammonium bicarbonate were added and then fully mixed.
(3) Different ratios of the pore-forming agent NH_4_HCO_3_ were added to the mixed raw materials, and the mixture was fully mixed. Then, pellets with a net weight of approximately 10 g were prepared under a pressure of 150 MPa. The prepared pellets are shown in Figure 2.
(4) The pellet sample was placed in a vacuum tube furnace, and the weight change in real-time was recorded. The pressure was lowered to 10 Pa by applying vacuum, and the pellet sample was heated from 100 °C to 1400 °C at a heating rate of 2 °C/min. Then, the weight loss curve of the pellet sample under the reaction conditions was obtained.

The weight loss curve of the pellet was transformed into the reduction conversion curve of magnesium, and the reduction conversion α was solved using Equation (1).

(1) $$\alpha =\frac{M_{before}-M_{after}}{M_{before}\times \omega \%}$$

where *M_before_* is the net weight of pellets without the pore-forming agent before the reaction, g; *M_after_* is the weight of residual pellets and reduced slag after the reaction, g; furthermore, ω is the theoretical content of magnesium in pellets without the pore-forming agent prior to the reaction, %.

**Figure 3:**
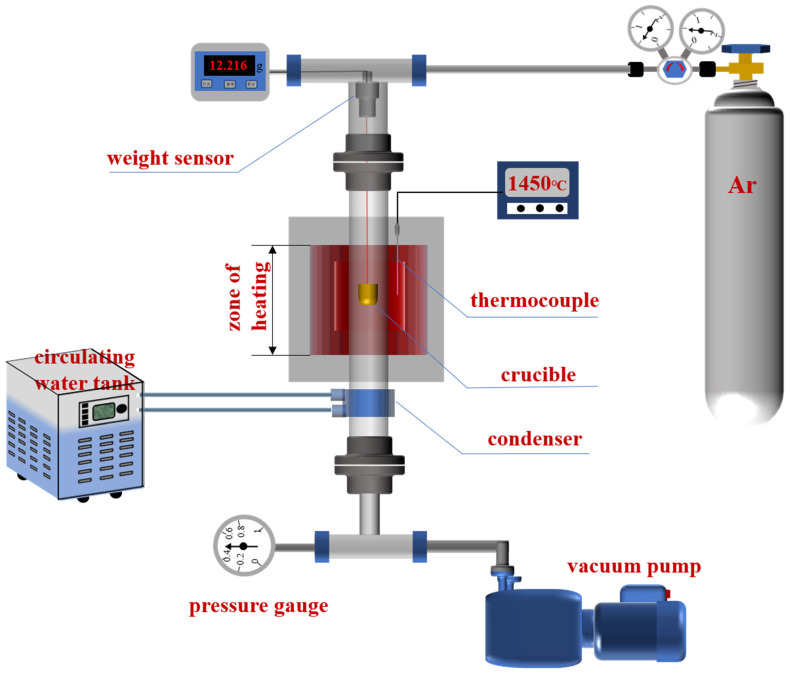
Working principle of the reaction device.

A thermogravimetric analysis (TGA) device, the core instrument used in the experiment, was developed by our research group, which could achieve the real-time measurement of weight changes in samples under high temperature and vacuum conditions. The principle of the device is shown in Figure 3.

## 3. Results and Discussion

During the heating of the sample, the gas molecules produced by the pyrolysis of pore-forming agent particles escape from the pellets. A cavity is generated in situ in the pore-forming agent particles, and the pore-forming agents uniformly distributed in the pellets are connected with each other. After the gas molecules escape, a cavity with high connectivity is formed inside the pellet, i.e., the three-dimensional cavity channel uniformly distributed inside the pellet can be used as the channel for the escape of magnesium vapor, reducing the resistance of magnesium escape, improving the efficiency of magnesium production, and improving the smelting efficiency.

### 3.1. Effect of Pore-Forming Agent on Pellet Density and Porosity

**Figure 4:**
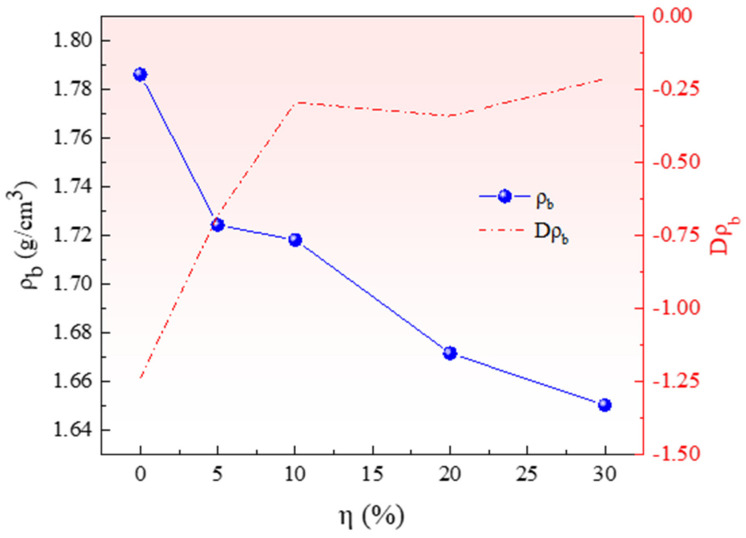
Relationship between pellet density and ratio of pore-forming agent.

The overall density ρ_b_ of pellets decreased from 1.79 g/cm^3^ to 1.65 g/cm^3^ with the increase of the ratio of pore-forming agent η from 0 to 30% due to the different densities of pore-forming agent and raw materials. The diameter and height of pellets after molding and demolding were measured using a Vernier caliper, D_b_ and H_b_, respectively. Through analysis and calculation, a relationship between the ratio of pore-forming agent and the change in pellet density was obtained, as shown in Figure 4.

Figure 4 shows that the pellet density ρ_b_ decreases with the increase in the ratio of pore-forming agent η because the bulk density prepared by NH_4_HCO_3_ under 150 MPa forming pressure is less than that of the pellet without a pore-forming agent. The curve of the change rate of pellet density Dρ_b_ shows that the addition of pore-forming agents from 0 to 10% significantly affects the pellet density. When the addition ratio is more than 10%, the density change rate of pellets decreases but the pellet density is infinitely close to the NH_4_CO_3_ pellet density.

**Figure 5:**
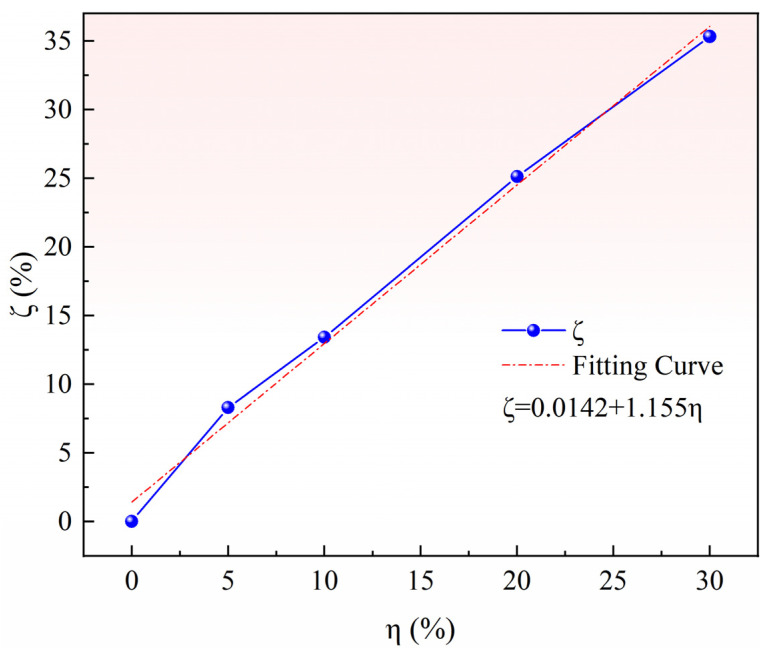
Relationship between the porosity and dosage of pore-forming agent.

The pore-forming agent in the pellets decomposes at high temperatures to generate carbon dioxide and ammonia, which escape from the pellets to form voids. Airflow gradually occurred with increasing gas pressure, forming porous channels after penetrating the voids. The pore-forming rate of pellets is closely related to the ratio of the pore-forming agent. The pore-forming rate of pellets is the volume ratio of the pore-forming agent in the whole pellet, and the relationship between the ratio of pore-forming agent η and pore-forming rate ζ can be obtained by conversion, as shown in Figure 5. The two show a linear relationship. After a linear fitting, the relationship between them is ζ = 0.0142 + 1.155η, and the correlation R^2^ is 0.99.

### 3.2. Reaction Characteristics of Pellets with Different Ratios of Pore-Forming Agents

**Figure 6:**
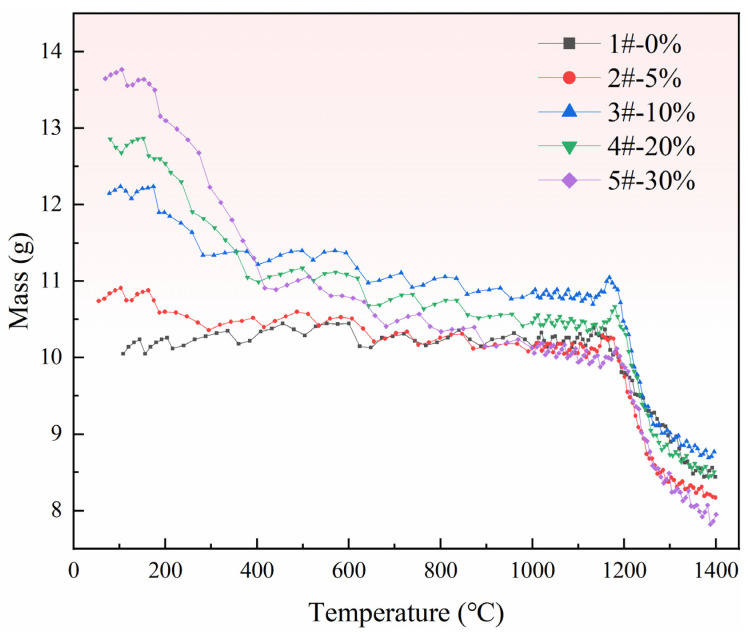
Mass change of pellets during heating.

Pellets with different ratios of pore-forming agents were reacted in a thermal analysis device with a vacuum of 10 Pa, and the temperature was increased from 100 °C to 1400 °C at a heating rate of 2 °C/min. The important changes in the samples during heating were recorded using a real-time weight monitoring system on the thermal analysis device, and the results are shown in Figure 6.

**Figure 7:**
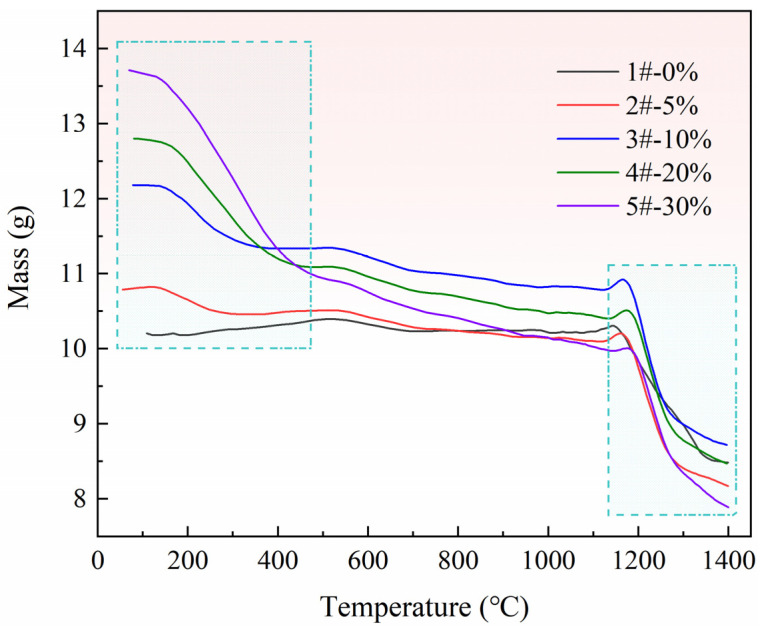
Mass change curves of samples after smoothing.

Affected by the accuracy and stability of the device itself, especially under vacuum conditions, the weight loss data are not very stable. However, the observation curve shows that the data change regularly. The LOWESS function in Origin software was used to smooth the curve, and the result is shown in Figure 7.

In Figure 7, curves 1–5# represent different ratios of pore-forming agent, which are 0, 5%, 10%, 20%, and 30%, respectively. There is an obvious mass change in curve 1#, indicating that the pellets without a pore-forming agent have an obvious chemical reaction during the entire heating process, i.e., the pellet mass changes because calcined dolomite is reduced by the silicon in the ferrosilicon to generate magnesium vapor, as shown in Reaction (2). In addition to curve 1#, two obvious mass changes are observed on curves 2~5# at the position of the dotted frame. The first step is due to the weight loss caused by the decomposition reaction of pore-forming agent NH_4_HCO_3_ at a lower temperature, and the pore-forming process of pellets is completed in this step, as shown in reaction (3). The reaction position of the second step is the same as that of curve 1#, and the magnesium is reduced at this stage.

(2) $$2{\left(\mathrm{CaO}\cdot \mathrm{MgO}\right)}_{\left(s\right)}{+\mathrm{Si}}_{\left(s\right)}\to 2\mathrm{CaO}\cdot {\mathrm{SiO}}_{2}{}_{\left(s\right)}{+2\mathrm{Mg}}_{\left(g\right)}$$

(3) $$NH_{4}HCO_{3(s)}\to CO_{2(g)}+H_{2}O_{\left(g\right)}+NH_{3(g)}$$

**Figure 8:**
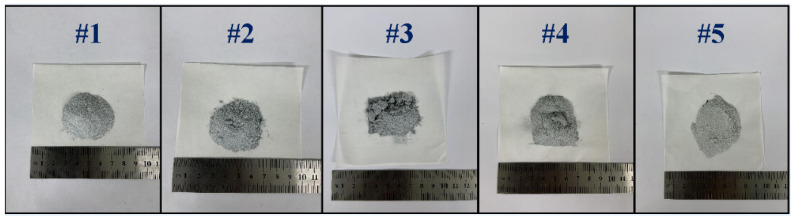
The residue after the reaction of different pellets.

The products obtained after the reaction were all in the powder state at room temperature, as shown in Figure 8, mainly because of the volume expansion caused by the phase transformation of calcium silicates during cooling. Moreover, each sample was involved in the reaction during heating, and the reaction residue nuclei were rarely found in the residue.

**Figure 9:**
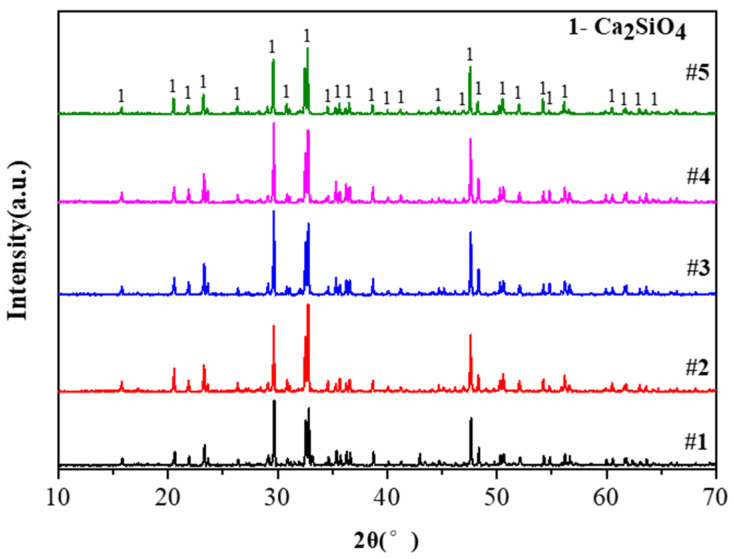
XRD patterns of residue after different pellet reactions.

Figure 9 shows the XRD patterns of residue powder after different pellet reactions. The residual component of the five samples after the reaction was 2CaO·SiO_2_, and no new composition was detected. This also indicates that the pore-forming agent turned into gas and escaped from the pellets after pore formation, and no new compositions were generated by reacting with the raw materials before and after pore-making. The choice of NH_4_HCO_3_ as a pore-forming agent will not have side effects.

### 3.3. Effect of Pore Formation Rate on Reduction Conversion of Pellets

**Figure 10:**
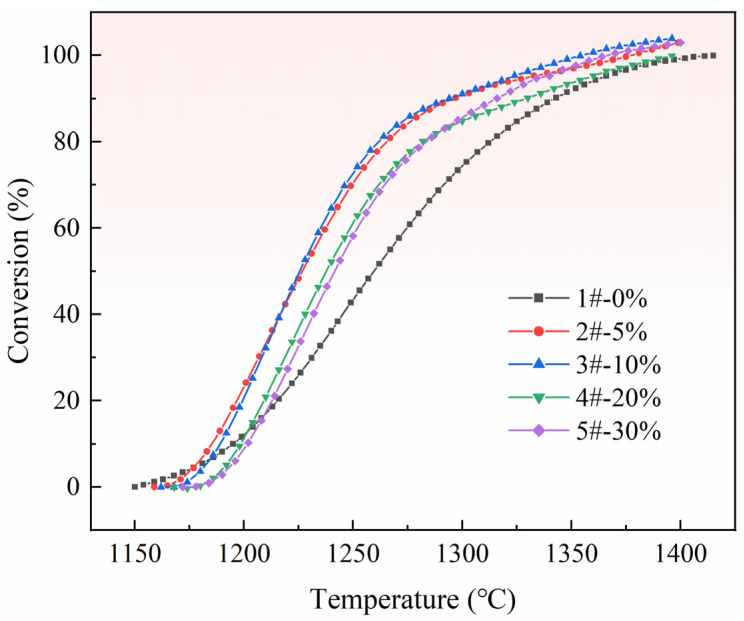
Change in reduction conversion of magnesium in pellets with different rations of pore-forming agent.

To evaluate the effect of porosity on the reaction conversion of ferrosilicon reduction of calcined dolomite in pellets, the second mass change in Figure 7 was selected as the research object, and using Equation (1), the weight loss of pellets was converted into the reduction conversion of magnesium. The results are shown in Figure 10.

As shown in Figure 10, the reduction conversion curves of the five sample pellets show an “S” trend overall, belonging to the typical solid-phase reaction type. The reaction characteristics of pellets (2# to 5#) with the pore-forming agent obviously changed after the completion of pore formation. When the ratio of pore-forming agent is 5%, the curve is steeper, and the reaction efficiency is faster than that of the pellets without a pore-forming agent. When the ratio of pore-forming agent increased to 10%, the curve trend is similar to that of 5%, and the steepness degree slightly increased. When the ratio of pore-forming agent increased to 20% and 30%, and the reduction conversion is lower than 80%, the trends of the four types of pellet samples with pore-forming agent (2# to 5#) curves are similar, but the starting points of the reaction are different. When the reduction conversion is more than 80% at the later stage of reduction reaction, the effect of pellet porosity on the reduction reaction is more obvious. The reduction conversion of 1# pellets remained constant, while the reduction rate of 2# to 5# pellets continued to show an increasing tendency. According to the above phenomenon, it can be concluded that an increase in the porosity in the pellets can effectively reduce the resistance of magnesium vapor generated by the reaction to escape from the pellets, thus improving the reduction efficiency.

**Figure 11:**
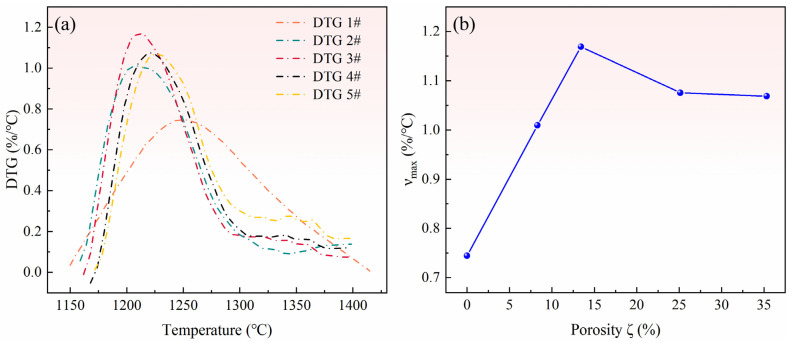
(**a**) Reduction rates of pellets with different porosities. (**b**) Relationship between pellet porosity and maximum reduction rate.

The reduction conversion of magnesium in pellets with different ratios of pore-forming agents can be used to obtain the rate of reduction reaction of each sample pellet at a high temperature, i.e., the rate of change of reduction conversion. Because all five samples were heated at the same rate, the rate of the reduction reaction could be obtained by differentiating the reduction conversion to temperature, as shown in Figure 11a.

Figure 11a shows that the reaction rate of pellets (2# to 5#) with pore-forming agent significantly increased compared with that of pellets (1#) without a pore-forming agent. When the pore-forming agent was 10%, the peak reaction rate was the largest, approximately 70% higher than that of pellets without a pore-forming agent. Figure 11b shows that with the increase in the ratio of pore-forming agent, the reduction rate first increased and then decreased. When the porosity increased from 0 to 15%, the instantaneous maximum change rate of reduction rate significantly increased. When the porosity increased from 15% to 35%, the instantaneous maximum rate decreased and became stable. Therefore, it can be inferred that an appropriate porosity of pellets can improve the reaction characteristics of pellets, and thus increase the reduction rate. However, when the porosity reaches a certain value, the increase in reduction rate is limited. It can be concluded that although the escape resistance of magnesium vapor decreases, the gas escape per unit flow channel also decreases, and the large porosity reduces the contact surface of reaction materials. Moreover, too much porosity also significantly reduces the heat transfer characteristics of pellets. Consequently, it can be seen from the experimental results that selecting the appropriate proportion of pore-forming agents can greatly improve the reduction efficiency, shorten the reduction time, reduce energy consumption, improve the production efficiency of magnesium, and achieve energy saving and emission reduction.

**Figure 12:**
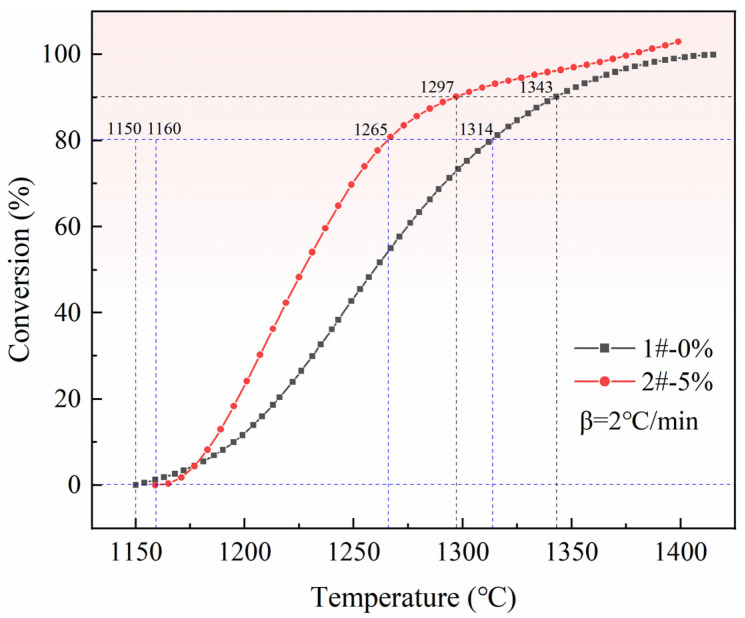
Comparison of reduction conversion between two pellets.

The reduction conversion of pellets without pore-forming agent and pellets with 5% pore-forming agent in the entire reaction process were compared and analyzed, and the results are shown in Figure 12.

Because the reaction was carried out at a constant heating rate of 2 °C/min, and the ratio of reaction time difference to the reaction temperature difference was the same, the increased amplitude in reduction conversion can be calculated from the temperature, as shown in Equation (4):

(4) $$\xi_{\left(\alpha \right)}=\frac{\left(T_{1-t}-T_{1-0})-(T_{2-t}-T_{2-0}\right)}{T_{1-t}-T_{1-0}}\times 100\%$$

where *T*_1−0_, *T*_1−*t*_, *T*_2−0_, and *T*_2−*t*_ represent the initial temperature of reaction of 1# and 2# pellets and the corresponding temperatures at a given reduction conversion, respectively; *ξ*(α) is the reduction efficiency increment for a specific reduction conversion.

According to Equation (4) and temperature data, the following conclusions are drawn:

(1) When the reduction conversion is 80%, the reduction efficiency of pellets with a 5% pore-forming agent is 36% higher than that of pellets without a pore-forming agent.
(2) When the reduction conversion is 90%, the reduction efficiency of pellets with a 5% pore-forming agent is 29% higher than that of pellets without a pore-forming agent.

Therefore, the addition of a pore-forming agent to pellets can produce porous pellets before the reduction reaction, thus achieving enhanced mass transfer. It effectively improves the diffusion rate of magnesium vapor in the raw pellet, shortens the time of chemical reaction, and improves the reduction efficiency of magnesium, thus shortening the smelting cycle, reducing the reduced energy consumption, improving the production efficiency, and reducing the production cost.

### 3.4. Characterization and Analysis of Crystallization Products

**Figure 13:**
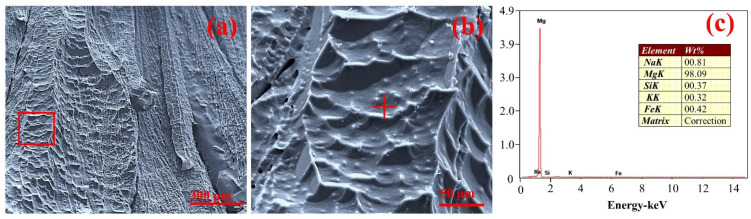
(**a**,**b**) Micromorphology of crystalline magnesium. (**c**) Energy spectrum analysis of crystalline magnesium.

Figure 13 shows the micromorphology and energy spectrum analysis of crystalline magnesium, which was collected at the condensation part of the lower end of the reactor.

A small amount of silver-white magnesium was collected at the lower part of the corundum tube inside the TGA furnace with the cooling water system. According to the SEM results of the samples shown in Figure 13a,b at different resolutions, under an absolute pressure of 10 Pa, the magnesium vapor will condense into crystalline magnesium when the temperature is lower than 500 °C [33,35]. The result of the energy spectrum in Figure 13c of the blank square in Figure 13a and the “+” region in Figure 13b shows that more than 98% of the crystalline magnesium was magnesium, but small amounts of Na, K, Si, and Fe were also observed. It is inferred that the Na and K present in the impurities are reduced by Si to form vapor. When the temperature of the water system is low enough, Na and K will condense and adhere to the surface of crystalline magnesium [32]. Additionally, all four elements may originate from the fly ash in the raw material. This is because, after repeated tests using the experimental device, there will always be some fly ash of the raw material adhered to the inner wall of the corundum tube and adsorbed on the surface of the crystallized magnesium when the magnesium vapor condenses.

## 4. Conclusions

By analyzing the factors affecting the mass transfer during magnesium production using the silicothermic method, a technological measure to enhance the mass transfer during magnesium production is proposed. By adding a proper ratio of pore-forming agent to the raw material to form porous pellets before the reduction reaction, the reactivity of the pellets can be improved, and the reduction efficiency can be increased.

Adding a pore-forming agent can appropriately increase the porosity, which is beneficial to the diffusion of solid-phase, enhances the mass transfer of magnesium vapor in the pellet, and greatly improves the reduction efficiency. When the reduction conversion is 80%, the reduction efficiency of pellets with a 5% pore-forming agent is increased by 36% compared with that without the pore-forming agent; when the reduction conversion is 90%, the reduction efficiency of pellets with a 5% pore former is improved by 29%.

The experiments showed that all the conversion curves showed a typical “S” shaped trend of solid-phase reactions, and with the increase of the pore-forming agent, the instantaneous maximum reduction rate first increases to the highest and then begins to decrease, and finally remains stable. Therefore, selecting the appropriate ratio of pore-forming agent can greatly improve the reduction efficiency, reduce the reaction time, reduce energy consumption, improve the efficiency of magnesium production, and realize energy saving and emission reduction.

## Figures and Tables

**Table 1:** Composition and calcining index of calcined dolomite (wt%).

Composition	MgO	CaO	Al_2_O_3_	Fe_2_O_3_	K_2_O	Na_2_O	CaF_2_	Si	Fe	Other	CO_2_	Hydration Activity (g)
Calcined dolomite	22.13	30.12	0.05	0.05	0.02	0.01				0.58	47.04	30.75
Ferrosilicon								76.10	19.69	4.21		
Fluorite				2.18	0.02	0.01	93.06			4.73		

## Data Availability

Data can be obtained from the corresponding author.
